# DIFFERENCES IN PHYSICAL PERFORMANCE MEASURES BETWEEN BLACK AND WHITE OLDER ADULTS

**DOI:** 10.1093/geroni/igad104.2455

**Published:** 2023-12-21

**Authors:** William Windham, B Gwen Windham, Hunter Sylvester, Priya Palta, Laura Skow, A Richey Sharrett, Anna Kucharska-Newton, Michael Griswold

**Affiliations:** University of Mississippi School of Medicine, Jackson, Mississippi, United States; UMMC-The MIND Center, Jackson, Mississippi, United States; UMMC-The MIND Center, Jackson, Mississippi, United States; UNC Gillings School of Global Public Health, Chapel Hill, North Carolina, United States; Johns Hopkins Bloomberg School of Public Health, Baltimore, Maryland, United States; Johns Hopkins Bloomberg School of Public Health, Baltimore, Maryland, United States; UNC Gillings School of Global Public Health, Chapel Hill, North Carolina, United States; UMMC-The MIND Center, Jackson, Mississippi, United States

## Abstract

Older Black adults are reported to have poorer physical function relative to White older adults. Cardiovascular risk factors and disease are associated with physical function and are more prevalent in Black adults. We examined objective physical function cross-sectionally using the Short Physical Performance Battery (SPPB, scored 0-12; 0.5 is considered clinically meaningful) in the Atherosclerosis Risk in Communities Study among Black and White participants during Visit 5 (2011-2013). Marginal standardization following generalized linear modelling (log-link, negative-binomial) was used to investigate whether cardiovascular features (hypertension, diabetes, BMI, smoking, heart disease, heart failure, stroke) helped explain race differences by comparing unadjusted (total effect) versus adjusted (direct effect) results. Among 5,836 participants (>65 years, mean age=75.9, 42% men, 22% Black), Black participants scored 1.3 points lower than White participants; unadjusted: 8.2 versus 9.5; relative difference of 0.86 (0.84, 0.88). After accounting for cardiovascular features, Black participants scored 1.4 points lower than White participants; adjusted: 8.2 versus 9.6; relative difference of 0.85 (0.84, 0.87). Cardiovascular risk factors and disease alone did not appear to account for substantial cross-sectional physical performance race differences in this population. Future research should examine additional factors potentially contributing to observed functional differences typically attributed to race such as cumulative vascular risk factors, geographic, social, cultural, and other systemic influences.

